# Interaction between human *APOE* polymorphisms and sex differences in hippocampal mitochondrial functions in lean and obese mice

**DOI:** 10.1002/alz70855_102266

**Published:** 2025-12-23

**Authors:** Zoe Paxhia‐Poppaw, Ushodaya Mattam, Brook Hemmelgarn, Noble Kumar Talari, Karthickeyan Chella Krishnan

**Affiliations:** ^1^ University of Cincinnati, Cincinnati, OH, USA

## Abstract

**Background:**

Alzheimer's disease (AD) is a neurodegenerative disease that affects 6.5 million Americans aged 65 or older. Of the 6.5 million Americans with AD, 4.2 million of them are female^,^ demonstrating that females are at higher risk for AD than males. One genetic risk factor for AD is *APOE* genotype. Apolipoprotein E (APOE) has three predominant alleles in humans: *APOE2, APOE3*, and *APOE4*. Individuals that are homozygous for *APOE4* have up to a 15‐fold increase in AD risk compared to *APOE3* homozygous individuals. A third, modifiable risk factor for AD is diet. A high fat, ‘Western’ style diet has been shown to be associated with AD.

**Method:**

12‐week‐old human *APOE3* and *APOE4* knock‐in mice of both sexes were fed a standard chow or Western diet for 16 additional weeks (total 6 months old) to investigate the effects of diet, sex, and *APOE* genotype. Hippocampus were isolated and processed for mitochondrial frozen respirometry and immunoblotting.

**Results:**

When fed a chow diet, there were no differences in respiration capacities between the *APOE3* and *APOE4* genotypes within the sexes. Chow‐fed females of both genotypes displayed significantly decreased mitochondrial complex I‐ and IV‐ dependent respiration capacities in the hippocampus compared to chow‐fed males. On a Western diet, *APOE4* males displayed significantly decreased mitochondrial complex I‐ and IV‐ dependent respiration capacities as well as significant decreases in all electron transport chain complex proteins compared to *APOE3* males.

**Conclusions:**

Our findings suggest that female *APOE4* KI mice exhibit reduced mitochondrial function under chow‐fed conditions compared to male *APOE4* KI mice, while a Western diet leads to mitochondrial maladaptation in male *APOE4* KI mice. A Western diet in female mice is likely to further exacerbate the observed sex differences in mitochondrial function, resulting in a more significant decrease in respiration capacities in *APOE4* females compared to *APOE3* females and/or *APOE4* males. Further research is needed to examine the effects of a Western diet on mitochondrial function in *APOE4* female mice.

